# Correlation between concentrations of salivary and gingival crevicular fluid inflammatory cytokines in patients with gingivitis and periodontitis and healthy individuals

**DOI:** 10.34172/japid.025.2244

**Published:** 2025-03-04

**Authors:** Ferya Azami, Vahid Esfahanian, Mahsa Ahmadi Shadmehri, Sami Rafiei

**Affiliations:** ^1^Department of Periodontics, Faculty of Dentistry, Isfahan (Khorasgan) Branch, Islamic Azad University, Isfahan, Iran

**Keywords:** Gingivitis, Interleukin-1β, Periodontitis, Tumor necrosis factor-α

## Abstract

**Background.:**

This study compared tumor necrosis factor-α (TNF-α) and interlukin-1β (IL-1β) concentrations in the saliva and gingival crevicular fluid (GCF) of patients with gingivitis and periodontitis and healthy individuals.

**Methods.:**

In this study, 45 patients (n=15 in each group) were selected, and the concentrations of inflammatory cytokines TNF-α and IL-1β in their salivary and GCF samples were measured using the ELISA method. Shapiro-Wilk test, one-way analysis of variance, post hoc Bonferroni test, and Pearson’s correlation coefficient were used to analyze the data.

**Results.:**

According to the results of the post hoc Bonferroni test, the mean salivary and GCF levels of TNF-α and IL-1β in healthy individuals were significantly lower than those with periodontitis (*P*<0.05) and gingivitis (*P*<0.05). Also, the mean levels of TNF-α and IL-1β in the saliva and GCF of patients with periodontitis were significantly higher than patients with gingivitis (*P*<0.05).

**Conclusion.:**

Based on the findings of the study, saliva, like GCF, can be considered a source for monitoring the markers related to periodontal disease. However, more uniformity was observed in GCF than in saliva in terms of alignment of markers secretion.

## Introduction

 Periodontitis, a prevalent inflammatory ailment, is driven by the interplay of pathogenic bacteria and host response.^1,2^ These bacteria induce immune responses, leading to the secretion of cytokines in the microenvironment around the alveolar bone. Notably, tumor necrosis factor-α (TNF-α) and interlukin-1β (IL-1β), secreted by monocytes-macrophages, play a crucial role in the degradation of periodontal tissues.^3,4^ TNF-α, an acute inflammatory mediator, aids leukocyte extraction, enhances vascular permeability, and induces osteoclast activation in response to bacterial lipopolysaccharides (LPS).^5-7^Similarly, IL-1β, a proinflammatory cytokine, stimulates T lymphocytes, cytokines, and B lymphocytes and promotes osteoclast differentiation, contributing to tissue destruction.^8-11^ Gingival crevicular fluid (GCF) is a reservoir of biomarkers associated with periodontal diseases, serving as a diagnostic tool for disease assessment.^12^

 Due to its rapid, easy, and non-invasive collection, saliva is a promising diagnostic fluid for monitoring periodontal diseases, presenting an alternative to GCF.^13^ Previous studies have explored biomarker variations in saliva, aiming to identify accurate markers for assessing periodontal status.^14^ This study seeks to explore the concentrations of inflammatory cytokines TNF-α and IL-1β in the saliva and GCF in healthy individuals and those with gingivitis and individuals with stage 2-3 periodontitis, examining their potential as diagnostic indicators.^1,13^

## Methods

 Forty-five patients (15 patients in each study group) referred to the Periodontology Department were included in this study. The patients’ conditions were assessed based on clinical attachment loss (CAL), bleeding on probing (BOP), and pocket depth (PD), and the inclusion criteria were as follows:

- Normal gingival color, no BOP in more than one site, and no proximal CAL for healthy individuals
- BOP in at least five sites, with PD ≤ 4 mm and no CAL for patients with gingivitis
- Periodontal PD ≥ 5 mm with loss of attachment of 2 mm and more, at least in three sites for patients with stages 2 and 3 periodontitis

Patients with a history of diabetes and pregnancy, use of antibiotics or anti-inflammatory drugs and drugs increasing the gingival volume, such as cyclosporine in the last three months, immune system disorders, smoking and using alcohol or any drug addiction, and a history of periodontal treatment in the previous six months were excluded. Before obtaining consent, patients were informed about the objectives of this study.

###  Collection of GCF samples 

 GCF samples were collected using a paper point from the gingival crevice or pocket. The selected areas were rinsed, dried, and isolated with a cotton roll. The paper points were placed in the gingival groove in the proximal areas, remained in place for two minutes, and then removed. The paper points were placed in a microtube, refrigerated at -5‒0 ºC, and then sent to the laboratory.

###  Collection of salivary samples 

 To collect salivary samples, the patients were asked to wash their mouths with water. Then, 5 mL of their unstimulated saliva was collected in a sterile tube. Test tube coding was used to blind the study after sampling. The collected saliva was transferred to the refrigerator and stored at -5‒0 ºC until delivery to the laboratory.

###  ELISA analysis

 In the present study, the ELISA test was conducted according to the manufacturer’s kit instructions. Salivary and GCF samples and 100 µL of standards with different concentrations were poured into standard and serum wells, respectively. 50 µL of anti-interleukin-1β and TNF-α antibodies conjugated to biotin were added to the wells. The wells’ surface was covered and incubated for 2 hours at room temperature (15‒18 ºC). The wells’ solution was emptied and washed six times with a wash buffer. 100 µL of streptavidin-HRP solution was added to all wells. The surface of the wells was covered and incubated for 1 hour at room temperature (15‒18 ºC). The wells’ solution was emptied and washed six times with a wash buffer. 100 µL of TMB solution (methyl benzidine) was added. The microplate was incubated for 10 minutes at room temperature in a dark place. 100 µL of stop solution was added, and the optical absorption of all the wells was read at 450 nm by an ELISA reader.

###  Data analysis 

 Analysis was conducted at both descriptive and inferential levels. Mean and standard deviation indices and statistical graphs were used at the descriptive level, one-way ANOVA, post hoc Bonferroni test, and Pearson’s correlation coefficient were used at the inferential level. The tests were performed at a 5% error level using SPSS 22.

## Results

 The variance analysis test showed a significant difference between the mean salivary and GCF levels of TNF-α and IL-1β in the three groups under study. According to the post hoc Bonferroni test results, the mean salivary and GCF levels of TNF-α and IL-1β in healthy individuals were significantly lower than those with periodontitis and gingivitis. Also, the mean levels of TNF-α and IL-1β in the saliva and GCF of patients with periodontitis were significantly higher than in patients with gingivitis (Table 1).

 Pearson’s correlation coefficient showed no significant relationship between TNF-α and IL-1β concentrations in the saliva and GCF of patients with gingivitis, periodontitis, and healthy individuals. Pearson’s correlation coefficient also showed a direct and significant relationship between TNF-α and IL-1β salivary and GCF concentrations in patients with periodontitis or gingivitis. In contrast, this relationship was not significant in healthy individuals (Table 2).

 Pearson’s correlation coefficient showed a direct and significant relationship between TNF-α concentration in saliva and GCF. There was also a direct and significant relationship between IL-1β concentrations in the saliva and GCF (Table 2).

 Based on Pearson’s correlation coefficient and Table 3, there was no significant relationship between IL-1β and TNF-α concentrations in the saliva and GCF of patients with gingivitis and periodontitis and healthy individuals. In contrast, a significant and direct relationship was observed between the concentration of IL-1β and TNF-α in the GCF of healthy individuals.

 Pearson correlation coefficient showed that, in general, there is no significant relationship between TNF-α and IL-1β concentrations in the saliva of patients with gingivitis and periodontitis. In contrast, there was a direct and significant relationship between the concentration of TNF-α and IL-1β in the total saliva of patients. The results of Pearson’s correlation coefficient test showed a direct and significant relationship between IL-1β and TNF-α concentrations in saliva and IL-1β and TNF-α concentrations in GCF.

**Table 1:** Mean and SD of TNF-α and IL-1β concentrations (EU/mL) in the saliva and gingival crevicular fluid of patients with gingivitis and periodontitis and healthy individuals

**Cytokine **	**Sample **	**Healthy individuals**	**Gingivitis**	**Periodontitis**	***P*** ** value**
TNF-α	Saliva	2.88 ± 0.48	23.56 ± 8.70	46.19 ± 13.71	< 0.001
	GCF	12.85 ± 1.79	3.36 ± 17.53	26.51 ± 5.50	< 0.001
IL-1β	Saliva	107.62 ± 19.28	285.55 ± 82.34	362.67 ± 64.87	< 0.001
	GCF	5.84 ± .95	37.44 ± 6.24	110.33 ± 25.50	< 0.001

**Table 2:** Pearson’s correlation coefficient between TNF-α and IL-1β concentrations in the saliva and GCF

**Cytokine type**	**Healthy individuals**	**Gingivitis**	**Periodontitis**	**All patients**	**All individual**
TNF-α	0.144- = r	r = - 0.005	= 0.204 r	0.545 = r	0.762 = r
	*P* = 0.304	0.493 = *P*	*P* = 0.233	0.001 = *P*	0.001 *P* <
IL-1β	0.087 - = r	0.408 = r	0.204 - = r	0.407 = r	0.739 = r
	= 0.757 *P*	0.131 = *P*	0.465 = *P*	0.026 = *P*	0.001 *P* <

**Table 3:** Pearson’s correlation coefficient between TNF-α and IL-1β concentrations in saliva and GCF

**Sample **	**Healthy individuals**	**Gingivitis**	**Periodontitis**
Saliva	0.097- = r	r = 0.354	= 0.094 r
	*P* = 0.731	0.196 = *P*	*P* = 0.739
GCF	0.535 = r	0.351 = r	0.169 - = r
	*P* = 0.040	0.200 = *P*	0.547 *= P*

## Discussion

 Inflammatory biomarkers, crucial for diagnosing periodontal diseases, are increasingly detected in the saliva. Biomarker studies aid in identifying sensitive patients and serve as indicators for treatment completion.^15^ Given the pivotal role of inflammatory cytokines in periodontal diseases and the conflicting study results on their elevation and association with clinical conditions, our study aimed to concurrently assess TNF-α and interleukin-1β levels in the saliva and GCF of patients with periodontitis, gingivitis, and healthy individuals.

 The findings of this study revealed that the mean concentration of TNF-α in the saliva and GCF was higher in patients with periodontitis compared to the gingivitis group and healthy individuals. Although statistical tests indicated a direct and significant relationship between TNF-α levels in the saliva and GCF, no significant correlation was observed in healthy individuals. Afacan et al’s^16^ study on inflammatory cytokines in the saliva and GCF demonstrated elevated TNF-α levels in GCF, positively correlating with clinical parameters with the highest values in the periodontitis group, aligning with our results.

 TNF-α, known to increase PGE2 concentration and activate osteoclasts, is expected to be higher in patients with periodontitis than in the gingivitis and healthy groups. However, studies by Yousefimanesh et al^17^ and Teles et al^18^ did not find significant differences in TNF-α levels in saliva between chronic periodontitis patients and healthy individuals, possibly due to age and gender matching or saliva collection methods. Our study confirmed significantly lower TNF-α GCF levels in healthy individuals compared to patients with periodontitis and higher levels than in those with gingivitis, consistent with Gokul et al’s^19^ findings.

 Similarly, the mean level of IL-1β in the saliva and GCF of patients with periodontitis was higher than in the gingivitis group and healthy individuals. Elevated IL-1β levels in the saliva of patients with gingivitis and periodontitis, compared to the healthy group, highlight the cytokine’s role in gingival and periodontal inflammation, supporting previous studies. However, conflicting results from Wu et al^20^ and Teles et al^18^ showed no significant differences in salivary IL-1β levels between chronic periodontitis patients and healthy individuals, potentially influenced by saliva collection methods and exclusion criteria such as not having systemic and inflammatory diseases, etc., which may lead to the involvement of confounding factors in determining the IL-1β level and thus change in the results of the study.

 Ebersole et al^21^ and Ramseier et al^22^ reported higher IL-1β salivary levels in periodontitis than in gingivitis, consistent with our study. Yücel et al^13^ observed varying IL-1β concentrations in GCF between groups, with higher rates in the gingivitis group than in the chronic periodontitis group, different from our results, indicating higher levels in patients with periodontitis. Some studies have demonstrated fluctuations in IL-1β salivary levels during periodontitis, particularly in gingivitis, over varying timeframes.^14^ Belstrøm et al^14^ found that intentionally avoiding oral hygiene for 10 days led to significant changes in IL-1β levels at different points. In experimental gingivitis, IL-1β levels decreased by the tenth day but returned to baseline after 14 days of oral hygiene instructions. Kinney et al^23^ observed significant decreases in IL-1β levels in the periodontitis group at 8, 10, and 12 months, in the gingivitis group at 8 and 10 months, and in the healthy group at 10 months compared to baseline. Their results indicated higher IL-1β levels in the gingivitis group during the second month than in the periodontitis groups (mild and moderate to severe).

 In summary, it cannot be conclusively stated that the secretion of these markers was consistently higher within each group, leading to a significant threshold difference and simultaneous secretion. Their secretion and function appear to vary across different disease stages. Therefore, combining both patient groups or adding potentially at-risk healthy individuals for comprehensive group comparisons reveals significant correlations. Additionally, the secretion of these markers in GCF exhibited more uniformity than in saliva.

 This study uniquely addressed existing literature contradictions by concurrently assessing inflammatory cytokine concentrations in the saliva and GCF across individuals with gingivitis and periodontitis and those in a healthy state. Unlike previous studies, which often focused on specific comparisons, our nuanced exploration of different periodontitis stages enhances our understanding of cytokine dynamics in diverse conditions. This distinctive methodology significantly advances knowledge, providing a more thorough perspective on inflammatory marker interplay in periodontal diseases.

 While our study showed a correlation between inflammatory cytokine concentrations in the saliva and GCF in individuals with gingivitis, periodontitis, and healthy conditions, certain limitations should be noted. The relatively small sample size (45 patients, 15 in each group) may limit generalizability. Exclusion criteria, essential for controlling confounding factors, might introduce selection bias. The cross-sectional design hinders establishing causality, and a broader array of biomarkers relevant to periodontal diseases was not explored. Despite these constraints, our study contributes valuable information to the literature on periodontal disease markers in the saliva and GCF.

## Conclusion

 Saliva, much like GCF, functions as a suitable medium for extracting and monitoring markers associated with periodontal diseases. If findings from similar studies align with our current research, saliva may be considered a preferred medium for assessing TNF-α, while GCF may prove more effective for estimating interleukin-1β.

## Competing Interests

 The authors declare that they have no competing interests.

## Data Availability Statement

 Data is available on request.

## Ethical Approval

 Informed consent was obtained from all individual participants in the study.
